# Medical Botany—Mr. Frost

**Published:** 1831

**Authors:** 


### LXXV.

### MEDICAL BOTANY—MR. FROST.

WHEN we reflect on the benefits which Mr. Frost conferred on the profession of this country, by bringing BOTANY into notice and estimation, under great difficulties and with the most unwearied exertions, we cannot but think that he has been rather harshly used, however imprudent he may have been, in some respects, while director of the Medico-Botanical Society. Others are now reaping the advantage of his toils and labours, and we trust that a generous public and a liberal profession will yet make some amends for the injuries which Mr. Frost has sustained in his prospects, by patronizing his botanical lectures, which we have incidentally learnt he is about to re-commence in this metropolis.

